# Oxidized Phospholipids and Neutrophil Elastase Coordinately Play Critical Roles in NET Formation

**DOI:** 10.3389/fcell.2021.718586

**Published:** 2021-09-09

**Authors:** Takuto Tokuhiro, Akane Ishikawa, Haruka Sato, Shunya Takita, Ayuri Yoshikawa, Ryoko Anzai, Shinichi Sato, Ryohei Aoyagi, Makoto Arita, Takumi Shibuya, Yasuaki Aratani, Shigeomi Shimizu, Masato Tanaka, Satoshi Yotsumoto

**Affiliations:** ^1^Laboratory of Immune Regulation, School of Life Sciences, Tokyo University of Pharmacy and Life Sciences, Tokyo, Japan; ^2^Frontier Research Institute for Interdisciplinary Sciences, Tohoku University, Sendai, Japan; ^3^Division of Physiological Chemistry and Metabolism, Graduate School of Pharmaceutical Sciences, Keio University, Tokyo, Japan; ^4^Laboratory for Metabolomics, RIKEN Center for Integrative Medical Sciences, Yokohama, Japan; ^5^Cellular and Molecular Epigenetics Laboratory, Graduate School of Medical Life Science, Yokohama City University, Yokohama, Japan; ^6^Graduate School of Nanobioscience, Yokohama City University, Yokohama, Japan; ^7^Department of Pathological Cell Biology, Medical Research Institute, Tokyo Medical and Dental University, Tokyo, Japan

**Keywords:** neutrophil extra cellular traps, myeloperoxidase (MPO), neutrophil elastase (NE), oxidized phospholipid, neutrophil (PMN)

## Abstract

Neutrophil extracellular traps (NETs) are web-like structures consisting of decondensed chromatin DNA and contents of granules, such as myeloperoxidase (MPO) and neutrophil elastase (NE). NETs are usually released from neutrophils undergoing NETosis, a neutrophil-specific cell death mode characterized by the collapse and disappearance of cell membranes and nuclear envelopes. It is well known that production of reactive oxygen species (ROS) triggers NETosis and NET formation. However, details of intracellular signaling downstream of ROS production during NETosis and NET formation remains uncertain. Here, we demonstrated that the peroxidation of phospholipids plays a critical role in NETosis and NET formation induced by phorbol 12-myristate13-acetate (PMA) or immune complex *in vitro* and by lipopolysaccharide (LPS) *in vivo*. This phospholipid peroxidation is mediated by the enzymatic activity of MPO. On the other hand, NE, which was previously reported to be released from granules to cytosol by MPO during NET formation, is not required for either the peroxidation of phospholipids or the execution of NETosis, but contributes to chromatin decondensation and nuclear swelling independently of MPO-mediated oxidized phospholipids. Analysis of isolated nuclei clearly demonstrated that oxidized phospholipids and NE differently yet synergistically execute chromatin decondensation and nuclear swelling, and the subsequent release of nuclear contents. These findings indicate the dual roles of MPO in NETosis and NET formation, and provide new insight into the molecular mechanism of these phenomena.

## Introduction

Neutrophils are immune cells that have such morphological features as lobulated nuclei and azurophilic granules. In the event of bacterial or viral infection, neutrophils contribute to biological defense by phagocytosing or killing these pathogens. On the other hand, neutrophils are also reported to be associated with tumor formation and the deterioration of autoimmune disease (Jorch and Kubes, 2017).

Neutrophil extracellular traps (NETs) have been attracting attention in recent years. NETs are web-like structures released from neutrophils and consist of decondensed chromatin DNA and contents of granules such as myeloperoxidase (MPO) and neutrophil elastase (NE) (Papayannopoulos et al., 2010; Metzler et al., 2011, 2014). NETs are known to be released by stimulation, such as phorbol 12-myristate13-acetate (PMA) or ionomycin stimulation, or physiological substances, such as bacteria, viruses, or immune complexes (ICs) (Papayannopoulos, 2018). Although NETs are released from living neutrophils under certain circumstances (Clark et al., 2007; Pilsczek et al., 2010; Yipp et al., 2012), their formation is usually accompanied by cell death called NETosis, which is characterized by the collapse and disappearance of cell membranes and nuclear envelopes (Fuchs et al., 2007). NET components are able to sterilize pathogens trapped by the physical force of NETs (Papayannopoulos, 2018). However, in sharp contrast to their benefits against infectious diseases, it is reported that NETs are involved in the exacerbation of various diseases and medical condition, such as autoimmune diseases, thrombosis, and cancer metastasis (Jorch and Kubes, 2017). Therefore, the control of NET formation is expected to produce therapeutic effects on these diseases and medical conditions.

Several molecules are involved in the molecular mechanisms underlying NET formation. Two molecules are involved in chromatin decondensation, which is a prerequisite for effective NET formation. During NET formation, peptidyl arginine deiminase 4 (PAD4) is activated by calcium signaling and the activated PAD4 decondenses chromatin DNA by the citrullination of histone H3 (Wang et al., 2009). Mice lacking PAD4 are susceptible to bacterial infection (Li et al., 2010), whereas another work showed that PAD4 deficiency promotes anti-fungal immune response (Alflen et al., 2020). In addition to PAD4, NE is reported to be involved in chromatin decondensation as well. This decondensation mechanism by NE is derived from the degradative action of histones. The release of NE from azurophilic granules to cytoplasm is reported to be regulated by MPO in an enzymatic activity-independent manner (Metzler et al., 2014).

The molecular events related to the initial signaling pathway for NETosis and NET formation have been extensively studied. Some inducers of NETosis and NET formation, including PMA, stimulate reactive oxygen species (ROS) production through the activation of NADPH oxidase. The genetic deficiency of NADPH oxidase in mouse and human results in failure of NET formation (Fuchs et al., 2007; Rohm et al., 2014). Although a few studies have demonstrated that ROS production is required for PAD4 activation (Neeli et al., 2008; Rohrbach et al., 2012), details regarding the consequences of ROS production during NETosis and NET formation remain uncertain.

We have previously reported that several chemical compounds promote NETosis and NET formation by accelerating phospholipid peroxidation, suggesting the involvement of oxidized phospholipids in these events (Yotsumoto et al., 2017). In this study, we demonstrated that phospholipid peroxidation is involved in NETosis and NET formation induced by some stimuli widely used as NET inducers. This lipid peroxidation is catalyzed by MPO and its enzymatic activity. Lipid peroxidation is required for the execution of NETosis, and oxidized phospholipids and NE coordinately execute nuclear swelling and chromatin decondensation, which are required for NET formation. These findings provide new insight into the molecular mechanisms of NETosis and NET formation.

## Results

### Lipid Peroxidation Is Involved in NET Formation

**FIGURE 1:**
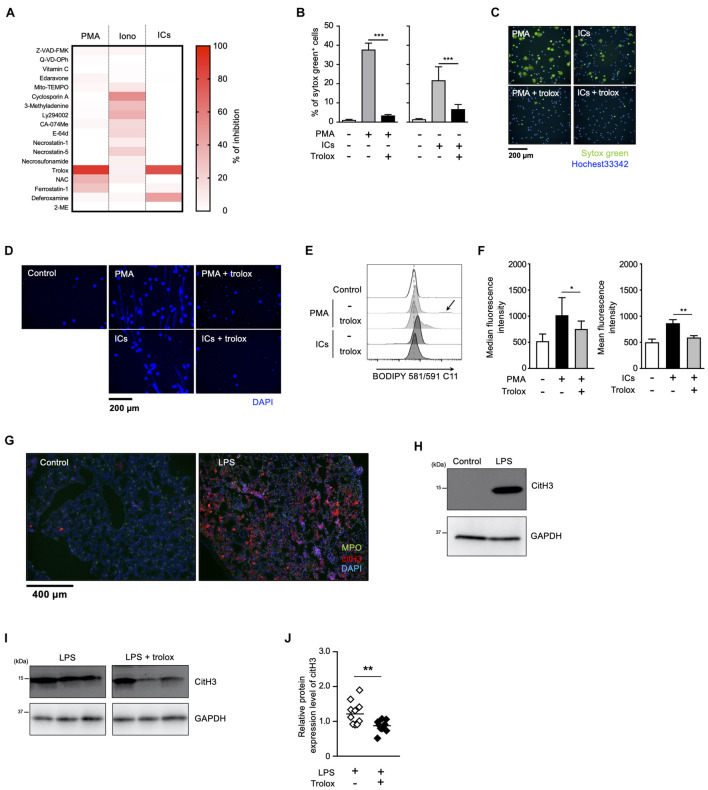
Lipid peroxidation is involved in NET formation *in vitro* and *in vivo*. **(A)** NETosis profiling of human neutrophils treated with cell death inhibitors. Peripheral blood neutrophils obtained from two healthy volunteers were stimulated with 50 nM PMA, 5 μM ionomycin, or 3 μg/mL ICs for 4 h in the presence or absence of various cell death inhibitors. Cells were stained with SYTOX Green and Hoechst 33342. The proportion of NETosis was determined by counting the number of SYTOX Green^+^ cells using a high-content analysis system. The degree of inhibition of each inhibitor is shown by a heatmap. Representative data of two independent experiments are shown. **(B–D)** Inhibitory effects of trolox on NETosis induction and NET formation induced by PMA or ICs. **(B,C)** Human neutrophils were stimulated with either 50 nM PMA or 3 μg/mL ICs for 4 h in the presence or absence of 1.6 mM trolox. The proportion of NETosis was determined using the same method as that in panel **(A)**. Average values and s.d. of triplicated samples in a single experiment are shown **(B)**. ***P < 0.005, one-way ANOVA. Representative images are shown **(C)**. Data are representative of two independent experiments. **(D)** Human neutrophils were stimulated with PMA or ICs in the presence or absence of 1.6 mM trolox. Cells were stained using DAPI and visualized by fluorescence microscopy. **(E,F)** Lipid peroxidation levels in neutrophils undergoing NETosis. Human neutrophils were stimulated with PMA or ICs for 1 h in the presence or absence of 1.6 mM trolox, and then incubated with BODIPY 581/591 C11. The accumulation of oxidized phospholipids was analyzed by flow cytometry. The higher peak is indicated by arrow. A representative flow cytometry plot is shown **(E)**. Average values of median fluorescent intensity with s.d. of twelve samples in four experiment are shown (**F**, left). Average values of mean fluorescent intensity with s.d. of triplicate samples in a single experiment are shown (**F**, right). *P < 0.05, **P < 0.01, one-way ANOVA. **(G,H)** C57BL/6 mice were administrated PBS (control) or 10 μg of LPS intranasally. Lungs were resected 24 h after injection. **(G)** Lung sections were analyzed by immunofluorescence analysis with anti-MPO antibody (MPO, green), anti-citH3 antibody (CitH3, red), and DAPI (blue). Data are representative of two independent experiments. **(H)** Western blot analysis of citH3 protein levels in lungs of mice treated as indicated. **(I,J)** Inhibitory effects of trolox on citH3 expression in lungs. Mice were injected intraperitoneally with 40 mg/kg trolox at 0 and 24 h prior to intranasal instillation of LPS. Twenty-four hours after instillation, the lungs were analyzed by Western blot with anti-citH3 antibody. Representative images of three mice in each group are shown **(I)**. CitH3 expression levels were normalized to GAPDH expression levels using Evolution Capt Software. Average values and s.d. of citH3 protein levels in each group (without trolox: *n* = 10, with trolox: *n* = 10) are shown **(J)**. **P < 0.01, unpaired *t*-test.

We have previously reported that sulfapyridine moiety-containing sulfa drugs, such as sulfasalazine (SSZ) and 4,4′-diaminodiphenyl sulfone (DDS), promote NETosis and subsequent NET formation in mouse and human neutrophils by accelerating the peroxidation of phospholipids (Yotsumoto et al., 2017). These findings suggest that phospholipid peroxidation may be involved in NETosis and NET formation promoted by inducers frequently used for these events. To characterize NETosis and NET formation, and to clarify in particular the relationship between lipid peroxidation and these events, we first tried to profile NETosis by using a series of inhibitors of various kinds of cell death. Human peripheral blood neutrophils were pretreated with each kind of cell death inhibitor or antioxidant, followed by stimulation with PMA, ionomycin or ICs for the induction of NETosis. The effects of these compounds on NETosis were evaluated using SYTOX Green, a membrane-permeable dye. As shown in Figure 1A, NETosis induced by either PMA or IC showed similar profiles, that is, trolox efficiently suppressed NETosis induced by both stimuli, whereas 2-mercaptoethanol had no effects on this cell death, indicating that lipid peroxidation is involved in NETosis induced by these stimuli (Figures 1A–C). Trolox is known to suppress ferroptosis by inhibiting lipid peroxidation (Dixon et al., 2012). However, in contrast to the effects of trolox, ferrostatin-1 (Fer-1) and deferoxamine (DFO), which are other inhibitors of ferroptosis showed weak suppressive activity on NETosis induced by PMA or ICs. Furthermore, we examined whether GPx4, a lipid ROS detoxifying enzyme, is involved in lipid peroxidation-dependent NETosis. This enzyme is reported to be responsible for the suppression of ferroptosis, and GPx4 deficiency results in the promotion of lipid peroxidation and ferroptosis (Yang et al., 2014). We isolated neutrophils from GPx4^flox/flox^ mice (Yoo et al., 2012) crossed with LysM-Cre mice, and stimulated these cells with PMA and DDS, an inducer for lipid peroxidation-dependent NETosis in mouse neutrophils (see below). As shown in (Supplementary Figure 1), levels of NETosis in GPx4-deficient neutrophils are comparable with that of WT neutrophils. These findings suggest lipid peroxidation in NET formation occurs by a completely different mechanism from ferroptosis. We also found that trolox showed strong inhibitory activity on NET formation associated with NETosis (Figure 1D). On the other hand, the profile of ionomycin-induced NETosis is completely different from that of PMA- or IC-induced NETosis, indicating that ionomycin-induced NETosis occurs in a lipid-peroxidation-independent manner. We also confirmed that trolox actually inhibited lipid peroxidation induced by PMA or ICs in neutrophils. Human peripheral blood neutrophils were treated with trolox and stimulated with PMA or ICs. Then, the neutrophils were treated with BODIPY 581/591 C11 and lipid peroxidation in the neutrophils was analyzed by flow cytometry. PMA or ICs induced lipid peroxidation in the neutrophils and this lipid peroxidation was completely suppressed by trolox (Figures 1E,F). Furthermore, we tried to observe the localization of oxidized lipids using BODIPY 581/591 C11 (Magtanong et al., 2019). Oxidized lipids were accumulated in plasma membrane and around nuclear region of mouse BM neutrophils undergoing NETosis (Supplementary Figure 2).

We next sought to examine whether lipid peroxidation is involved in NET formation *in vivo*. For this purpose, C57BL/6J mice were administrated 10 μg of LPS intranasally to induce NET formation in activated neutrophils infiltrating lungs. Twenty-four hours after the LPS administration, NET formation was clearly detected in lungs by immunofluorescence analysis for MPO, citrullinated histone H3 (CitH3), and DNA (Figure 1G). The increase in citH3 levels was confirmed by Western blotting with anti-citH3 antibody (Figure 1H). As shown in Figures 1I,J, intraperitoneal injection of trolox decreased the increased citH3 levels in lungs induced by LPS, indicating that lipid peroxidation is involved, at least, in part, in LPS-induced NET formation in lungs.

### Differential Contribution of MPO and NE in NETosis and NET Formation

**FIGURE 2:**
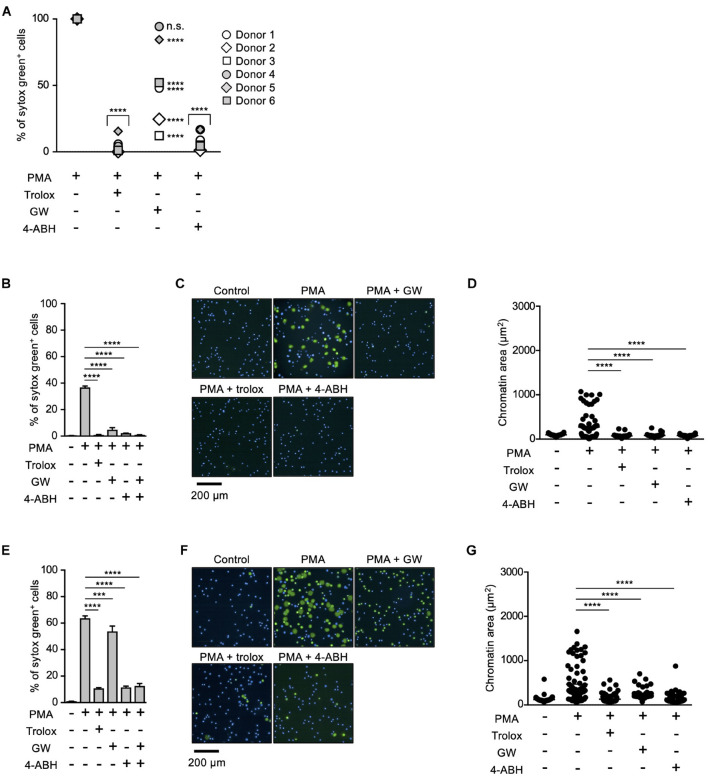
Differential contribution of MPO and NE in NETosis induction and NET formation. **(A)** Human neutrophils were isolated from six healthy donors. Neutrophils were stimulated with 50 nM PMA for 4 h in the presence or absence of 1.6 mM trolox, 500 μM 4-ABH, or 20 μM GW311616. Cells were stained with SYTOX Green and Hoechst 33342. The proportion of NETosis was determined using the same method as that in Figure 1A. n.s., not significant, ****P < 0.001, two-way ANOVA, compared with PMA-treated cell. **(B–G)** Effects of NE inhibitors on nuclear swelling in human neutrophils. Neutrophils were isolated from two healthy donors [a responder to the NE inhibitor in NETosis **(B–D)** and a non-responder to the NE inhibitor in NETosis **(E–G)**]. Neutrophils were stimulated with 50 nM PMA for 3.5 h in the presence or absence of 1.6 mM trolox, 500 μM 4-ABH, or 20 μM GW311616, followed by staining with SYTOX Green and Hoechst 33342. The proportion of NETosis was determined using the same method as that in Figure 1A. Average values and s.d. of triplicate samples in a single experiment are shown. ***P < 0.005, ****P < 0.001, one-way ANOVA **(B,E)**. Representative images of three independent experiments are shown **(C,F)**. The chromatin areas of 70–76 neutrophils are calculated in each condition using Image-J software. ****P < 0.001, one-way ANOVA **(D,G)**.

Myeloperoxidase and NE have been reported to play critical roles in NETosis and NET formation (Papayannopoulos et al., 2010; Metzler et al., 2011, 2014; Bjornsdottir et al., 2015). Thus, we next sought to reveal the relationship between these enzymes and lipid peroxidation in the execution of NETosis and NET formation. For this purpose, we first compared the effects of MPO or NE inhibitors on NETosis and NET formation with those of trolox in human neutrophils obtained from several healthy donors. As shown in Figure 2A, trolox and MPO inhibitors completely suppressed NETosis induced by PMA stimulation in neutrophils from all donors. On the other hand, the effects of the NE inhibitor varied among donors, suggesting that NE is not always necessary for the execution of NETosis in human neutrophils. We also assessed the effects of these compounds on NET formation by analyzing nuclear morphology using Operetta CLS High-Content Analysis system. In the case that the NE inhibitor suppressed NETosis, all inhibitors suppressed nuclear swelling, which is one characteristic of NET formation, as well (Figures 2B–D). Intriguingly, not only trolox and MPO inhibitors, but also the NE inhibitor significantly suppressed nuclear swelling even in the case that the NE inhibitor did not suppress NETosis (Figures 2E–G). These results indicate that NE is not always required for the execution of NETosis, but is actually involved in NET formation.

### MPO Plays a Critical Role in Lipid Peroxidation-Dependent NET Formation

**FIGURE 3:**
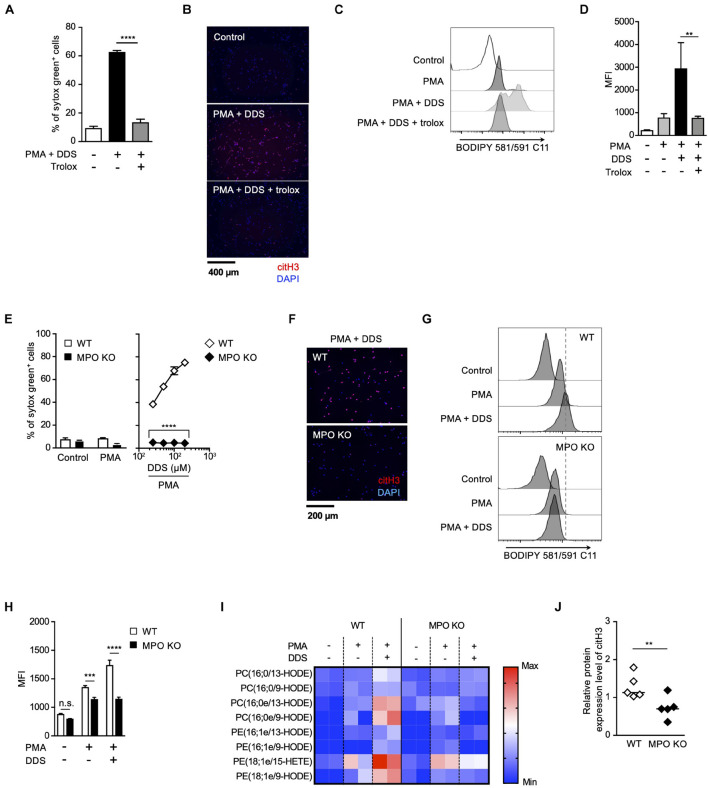
MPO plays a critical role in lipid peroxidation-dependent NET formation. **(A–D)** PMA and DDS induce lipid peroxidation-dependent NET formation in mouse bone marrow neutrophils. **(A,B)** Neutrophils were stimulated with 1 μM PMA and 200 μM DDS in the presence or absence of 400 μM trolox for 4 h. **(A)** The proportion of NETosis was determined using the same method as that in Figure 1A. Average values and s.d. of triplicate samples in a single experiment are shown. ****P < 0.001, one-way ANOVA, compared with PMA and DDS-treated cells. **(B)** NET formation was visualized by staining the cells for DNA (DAPI) and with anti-citH3 monoclonal antibody. Original magnification, ×20. Data are representative of two independent experiments. **(C,D)** Neutrophils were stimulated with 1 μM PMA and 200 μM DDS in the presence or absence of 400 μM trolox for 1.5 h. BODIPY 581/591 C11 was added. The accumulation of oxidized phospholipids was analyzed by flow cytometry. A representative flow cytometry plot is shown **(C)**. Average MFI values and s.d. of triplicate samples are shown. **P < 0.01, one-way ANOVA, compared with PMA and DDS-treated cells **(D)**. Data are representative of three independent experiments. **(E–J)** MPO is essential for lipid peroxidation-dependent NETosis induction and NET formation. WT- and MPO-deficient neutrophils were stimulated with PMA with or without DDS. NETosis induction **(E)** and NET formation **(F)** were evaluated as described above. Average values and s.d. of triplicate samples in a single experiment are shown. ****P < 0.001, unpaired *t*-test, compared with WT neutrophils. **(G,H)** WT or MPO-deficient mouse neutrophils were stimulated with 1 μM PMA with or without DDS. The accumulation of oxidized phospholipids was determined as described above. Average values and s.d. of triplicate samples in a single experiment are shown. ***P < 0.005, ****P < 0.001, n.s., not significant, one-way ANOVA **(E)**, unpaired *t*-test **(H)**, compared with WT neutrophils. Data are representative of three independent experiments. **(I)** Wide-targeted lipidomics of oxidized phospholipids. WT or MPO-deficient mouse neutrophils were stimulated with 1 μM PMA and 200 μM DDS for 1.5 h. Lipid was extracted from these neutrophils, and wide-targeted analysis was performed using an ACQUITY UPLC system coupled with a triple quadrupole MS. The degree of quantity of each oxidized phospholipid is shown by a heatmap. **(J)** NET formation is suppressed in lung of MPO-deficient mice. WT or MPO-deficient mice were administrated 10 μg of LPS intranasally. Twenty-four hours after administration, the lungs were analyzed by Western blots with anti-citH3 antibody. CitH3 expression levels were normalized to GAPDH expression levels using Evolution Capt Software. Average values and s.d. of citH3 protein levels in each group (WT: *n* = 5, MPO KO: *n* = 5) are shown. **P < 0.01, unpaired *t*-test.

Strong correlation between the effects of MPO inhibitor and trolox on the execution of NETosis and NET formation prompted us to speculate a causal relationship between MPO activation and lipid peroxidation in these events. To prove this hypothesis, we next examined whether MPO is involved in lipid peroxidation-dependent NET formation using MPO-deficient mice. We have previously reported that PMA and DDS stimulation induces lipid peroxidation-dependent NETosis and NET formation in mouse neutrophils, whereas PMA alone has little effects on inducing NETosis and NET formation in mouse neutrophils (Yotsumoto et al., 2017). Therefore, we used this system to examine the correlation between MPO and lipid peroxidation in mouse neutrophils. PMA and DDS stimulation induced lipid peroxidation and subsequent NETosis and NET formation in neutrophils from wild type (WT) mice, and these events were completely suppressed by trolox (Figures 3A–D), indicating that these stimuli induce lipid peroxidation-dependent NETosis and NET formation in mouse neutrophils. In sharp contrast, NETosis and NET formation by PMA and DDS stimulation were not observed in neutrophils from MPO^–/–^ mice (Figures 3E,F). We also investigated whether MPO is involved in lipid peroxidation induced by PMA and DDS by using BODIPY 581/591 C11. DDS significantly enhanced PMA-induced lipid peroxidation in neutrophils from WT mice but not in neutrophils from MPO^–/–^ mice (Figures 3G,H). We further compared the amount of oxidized phospholipids in WT or MPO^–/–^ neutrophils by lipidomics analysis. WT or MPO^–/–^ neutrophils were stimulated with PMA and DDS for 1.5 h, lipids were extracted, and wide-targeted analysis was performed using an ACQUITY UPLC system coupled with a triple quadrupole MS. The increase in the amount of oxidized phospholipids including 9-hydroxyoctadecadienoic acid (9-HODE) and 13-hydroxyoctadecadienoic acid (13-HODE) was observed in WT neutrophils but not in MPO^–/–^ neutrophils (Figure 3I). These results also indicate that MPO is key mediator of lipid peroxidation. We further checked the involvement of MPO in lipid peroxidation-dependent NET formation in lungs. As shown in Figure 3J, the levels of citH3 were significantly reduced in the lungs of MPO KO mice. Taken together, these data clearly indicate that MPO is involved in lipid peroxidation-dependent NET formation.

### Enzymatic Activity of MPO Is Required for Lipid Peroxidation-Dependent NETosis and NET Formation

**FIGURE 4:**
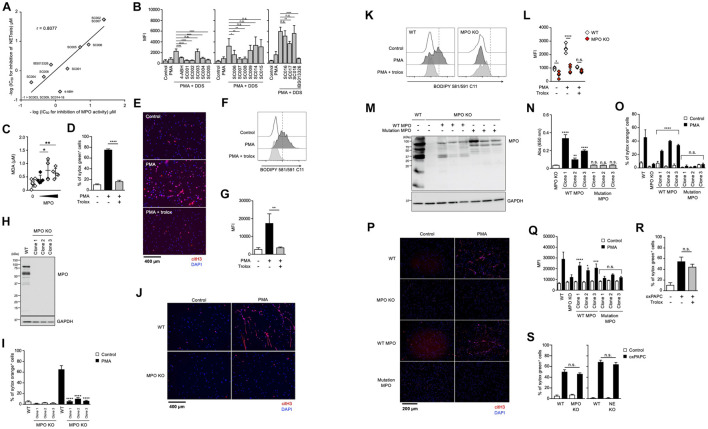
Enzymatic activity of MPO is required for lipid peroxidation-dependent NETosis induction and NET formation. **(A)** Correlation between MPO-inhibitory activity and NET-inhibitory activity of several hydrazine compounds. The inhibitory effects of each compound on enzymatic activity of MPO were evaluated as described in the Section “Materials and Methods.” Mouse neutrophils were stimulated with 1 μM PMA and 200 μM DDS for 3 h in the presence or absence of each hydrazine compound. The proportion of NETosis was determined using the same method as that in Figure 1A. IC_50_ of each compound for MPO-inhibitory activity and NET-inhibitory activity was plotted. **(B)** Mouse neutrophils were stimulated with 1 μM PMA and 200 μM DDS in the presence or absence of each hydrazine compound for 1 h. Then, BODIPY 581/591 C11 was added. The accumulation of oxidized phospholipids was analyzed by flow cytometry. Average MFI values and s.d. of triplicated samples are shown. *P < 0.05, **P < 0.01, ****P < 0.001, n.s., not significant, one-way ANOVA, compared with PMA + DDS-treated cells. Data are representative of three independent experiments. **(C)** Lipid peroxidation ability of MPO. Egg PC was incubated with MPO in the presence of H_2_O_2_. MDA levels were assessed by TBARS assay. Average values and s.d. of 4–7 samples in three experiments are shown. *P < 0.05, **P < 0.01, one-way ANOVA, compared with untreated PAPC. **(D,E)** dHL-60 cells were stimulated with 50 nM PMA in the presence or absence of 1.6 mM trolox for 4.5 h. The proportion of NETosis was determined using the same method as that in Figure 1A. ****P < 0.001, one-way ANOVA, compared with PMA-treated cells. Average values and s.d. of triplicate samples in a single experiment are shown **(D)**. NET formation was visualized by staining the cells with DAPI and anti-citH3 monoclonal antibody **(E)**. Original magnification, ×20. Data are representative of two independent experiments. **(F,G)** dHL-60 cells were stimulated with 0.1 μM PMA in the presence or absence of 1.6 mM trolox for 1 h. Then, BODIPY 581/591 C11 was added. The accumulation of oxidized phospholipids was analyzed using flow cytometry. A representative flow cytometry plot is shown **(F)**. Average MFI values and s.d. of triplicate samples are shown **(G)**. **P < 0.01, one-way ANOVA, compared with PMA-treated cells. Data are representative of three independent experiments. **(H–L)** MPO-deficient HL-60 cells were established by using CRISPR/Cas9. A representative immunoblot confirming the absence of MPO in HL-60 cells **(H)**. GAPDH was used as the loading control. WT- and MPO-deficient dHL-60 clones were analyzed for NETosis **(I)**, NET formation **(J)**, and lipid peroxidation **(K,L)**, as described above. *P < 0.05, ****P < 0.001, n.s., not significant, one-way ANOVA, compared with PMA-treated WT dHL-60 **(I,L)**. Data are representative of two independent experiments. **(M–Q)** MPO-deficient HL-60 cells were reconstituted with either WT MPO- or enzymatic-inactive MPO mutant protein. A representative immunoblot confirming the presence of WT- or mutant MPO_D__96__A_ in MPO-deficient HL-60 cells **(M)**. GAPDH was used as the loading control. Enzymatic activity of MPO was measured in MPO-deficient cells or MPO-deficient cells reconstituted with WT MPO or mutant MPO **(N)**. Analysis of NETosis induction **(O)**, NET formation **(P)**, and lipid peroxidation **(Q)** as described above. *P < 0.05, **P < 0.01, ***P < 0.005, ****P < 0.001, n.s., not significant, one-way ANOVA, compared with MPO-deficient HL-60 **(N)** and PMA-treated MPO-deficient dHL-60 **(O,Q)**. Data are representative of two independent experiments. **(R)** dHL-60 cells were stimulated with 100 μg/mL oxPAPC in the presence or absence of 1.6 mM trolox for 4 h. The proportion of NETosis was determined using the same method as that in Figure 1A. n.s., not significant, one-way ANOVA, compared with oxPAPC-treated dHL-60. Average values and s.d. of triplicate samples in a single experiment are shown. Data are representative of two independent experiments. **(S)** WT-, NE-deficient or MPO-deficient dHL-60 cells were stimulated with 100 μg/mL oxPAPC for 3.5 h. The proportion of NETosis was determined using the same method as that in Figure 1A. n.s., not significant, one-way ANOVA, compared with WT dHL-60. Average values and s.d. of triplicate samples in a single experiment are shown. Data are representative of two independent experiments.

It has been reported that MPO contributes to the release of NE from granules and the subsequent induction of NET formation in an enzymatic activity-independent manner (Metzler et al., 2014). Thus, we sought to clarify whether MPO enzymatic activity is required for lipid peroxidation-dependent NETosis or not. First, we analyzed the structure-activity relationship of MPO inhibitors in lipid peroxidation-dependent NETosis. The hydrazine moiety is the active center of some MPO inhibitors (Malle et al., 2007). We identified additional NETosis inhibitor (IBS013326) that has the hydrazine moiety by screening a compound library. Then, we investigated 15 structure-related hydrazine compounds (Supplementary Figure 3) in terms of correlation between suppressive activity on NETosis and inhibitory effects on the enzymatic activity of MPO. As shown in Figure 4A, there was a significant correlation between IC_50_ for the inhibition of NETosis and IC_50_ for the inhibition of MPO enzymatic activity in these compounds. In accordance with the good correlation between these two inhibitory activities, lipid peroxidation and inhibitory effect of MPO enzymatic activity are strongly correlated (Figure 4B), that is, compounds possessing inhibitory activity against NETosis suppress lipid peroxidation. These data strongly support that MPO enzymatic activity plays a critical role in the induction of lipid peroxidation-dependent NETosis.

We next investigated whether MPO possessed phospholipid oxidizing ability. For this purpose, the TBARS assay was used to evaluate lipid peroxidation ability with Egg PC as the substrate. When phosphatidylcholine was incubated with H_2_O_2_ in the presence of purified MPO, the amount of MDA (malondialdehyde), a marker of lipid peroxidation, increased depending on the concentration of MPO (Figure 4C). These results suggest that MPO enzymatic activity directly triggers lipid peroxidation during NETosis and NET formation.

We conducted another experiment to confirm the importance of MPO enzymatic activity in the execution of NETosis and NET formation. We used HL-60, a human promyelocytic leukemia cell line. HL-60 cells treated with dimethyl sulfoxide (DMSO) are known to differentiate into neutrophil-like cells. To confirm whether HL-60 cells differentiate into neutrophil-like cells, we analyzed HL-60 treated with DMSO. DMSO-treated cells showed partially lobulated nuclei, whereas untreated cells had round nuclei (Supplementary Figure 4a). Treatment of HL60 cells with DMSO for 7 days resulted in an increase in CD11b and CD16 cell surface expression (Supplementary Figure 4b). DMSO-treated HL-60 cells also highly expressed *peptidylarginine deiminase 4* (*PAD4*) and *p47phox* (*NCF1*), a subunit of NADPH-oxidase (Supplementary Figure 4c). DMSO-treated HL-60 cells produced ROS when treated with PMA (Supplementary Figure 4d), confirming that DMSO-treated HL-60 (dHL-60) cells possess several features of neutrophils. After differentiation, HL-60 cells underwent NETosis and formed NETs in a lipid peroxidation-dependent manner (Figures 4D–G). Using this cell line, we first established MPO-deficient HL60 clones by the CRISPR/Cas9 system. Mutation in *MPO* gene and deficiency of MPO protein in each clone were confirmed by DNA sequencing and Western blot analysis, respectively (Figure 4H). As shown in Figures 4I,J, NETosis and NET formation were not induced in MPO-deficient HL-60 cells. Consistent with these results, lipid peroxidation was markedly suppressed in MPO-deficient cells (Figures 4K,L). Then, we reconstituted HL-60 clones with either WT MPO or enzymatic-inactive mutant MPO, which has the mutation of aspartic acid to alanine mutation at amino acid position 96 (D96A) in the light chain (Figure 4M) (Shin et al., 2001). The enzymatic activity of MPO was recovered in cells reconstituted with WT MPO, but in cells reconstituted with mutant MPO (Figure 4N). The reconstitution with WT MPO, but not mutant MPO, resulted in recovery of HL-60 clones that underwent NETosis and NET formation (Figures 4O,P). Consistent with these results, lipid peroxidation occurred in WT MPO-reconstituted cells but not in mutant MPO-reconstituted cells (Figure 4Q). These data clearly indicate that MPO contributes to the execution of NETosis and NET formation through its enzymatic activity to oxidize phospholipids.

Given that MPO contributes to the execution of NETosis and subsequent NET formation by oxidizing phospholipids, the oxidized phospholipids presumably induce NETosis directly even in the absence of MPO. To prove this, we sought to examine whether oxidizing phospholipids induce NETosis in MPO-deficient HL-60 cells. As shown in Figure 4R, oxidized 1-palmitoyl-2-arachidonoyl-sn-glycero-3-phosphocholine (oxPAPC) induced NETosis, and trolox did not suppress this cell death. As expected, oxPAPC induced NETosis in MPO-deficient HL60 cells in the same manner as in WT HL-60 cells (Figure 4S).

Taken together, we conclude that MPO oxidizes phospholipids and subsequently induces NETosis and NET formation.

### NE Is Required for Not NETosis but NET Formation in dHL60 Cells

**FIGURE 5:**
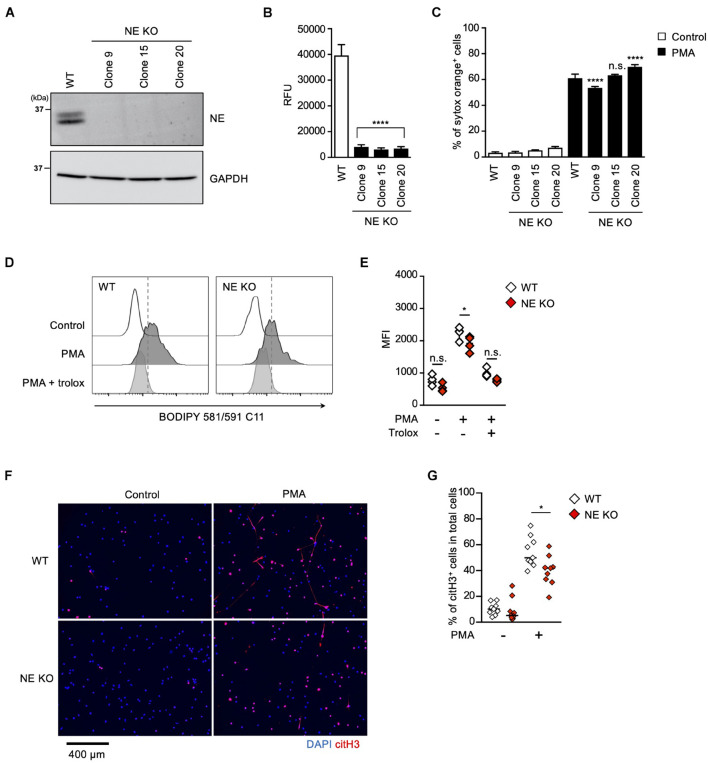
NE is required for not NETosis but NET formation. **(A–C)** NE-deficient HL-60 cells were established using CRISPR/Cas9. **(A)** A representative immunoblot confirming the deficiency of NE in HL-60 cells. GAPDH was used as the loading control. **(B)** NE-enzymatic activity was evaluated as described in the Section “Materials and Methods.” ****P < 0.001, one-way ANOVA, compared with cell lysate from WT dHL-60 cells. Average values and s.d. of triplicate samples in a single experiment are shown. **(C)** WT- or NE-deficient dHL-60 cells were stimulated with 50 nM PMA for 3.5 h. The proportion of NETosis was determined using the same method as that in Figure 1A. ****P < 0.001, n.s., not significant, one-way ANOVA, compared with PMA-treated WT dHL-60 cells. Average values and s.d. of triplicate samples in a single experiment are shown. **(D,E)** WT- or NE-deficient dHL-60 cells were stimulated with 50 nM PMA in the presence or absence of 1.6 mM trolox for 1 h. Then, BODIPY 581/591 C11 was added. The accumulation of oxidized phospholipids was analyzed by flow cytometry **(D)**. Average MFI values of triplicate samples are shown **(E)**. *P < 0.05, n.s., not significant, one-way ANOVA, compared with WT dHL-60. Data are representative of three independent experiments. **(F,G)** WT- or NE-deficient dHL-60 cells were stimulated with 50 nM PMA for 3.5 h. NET formation was visualized by staining the cells with DAPI and anti-citH3 monoclonal antibody **(F)**. Original magnification, ×20. Images are representative fields from three experiments. The proportion of citH3^+^ cells in fields was determined using Image-J software **(G)**. Average values of nine fields from three clones are shown. *P < 0.05, one-way ANOVA, compared with PMA-treated WT dHL-60.

To further explore the role of the MPO-NE axis in lipid peroxidation-dependent NETosis and NET formation, we established NE-deficient HL-60 cells (Figure 5A). In accordance with the deficiency of NE protein, intracellular NE activities were abolished in these NE-deficient clones (Figure 5B). However, PMA stimulation induced NETosis in the NE-deficient clones as efficiently as in parent cells (Figure 5C). In addition, the degree of lipid peroxidation induced by PMA was not affected by NE deficiency (Figures 5D,E). These data also support the idea that NE is not involved in lipid peroxidation-dependent NETosis. We further checked NET formation in NE-deficient HL-60 cells by evaluating immunostaining with anti-citH3 antibody. Although NE deficiency did not affect NETosis, the number of citH3-positive cells was significantly reduced in NE-deficient HL-60 cells (Figures 5F,G).

These results clearly indicate that NE is dispensable for lipid peroxidation-dependent NETosis, but is involved in NET formation.

### Oxidized Phospholipids and NE Coordinately Play Critical Roles in Chromatin Decondensation

**FIGURE 6:**
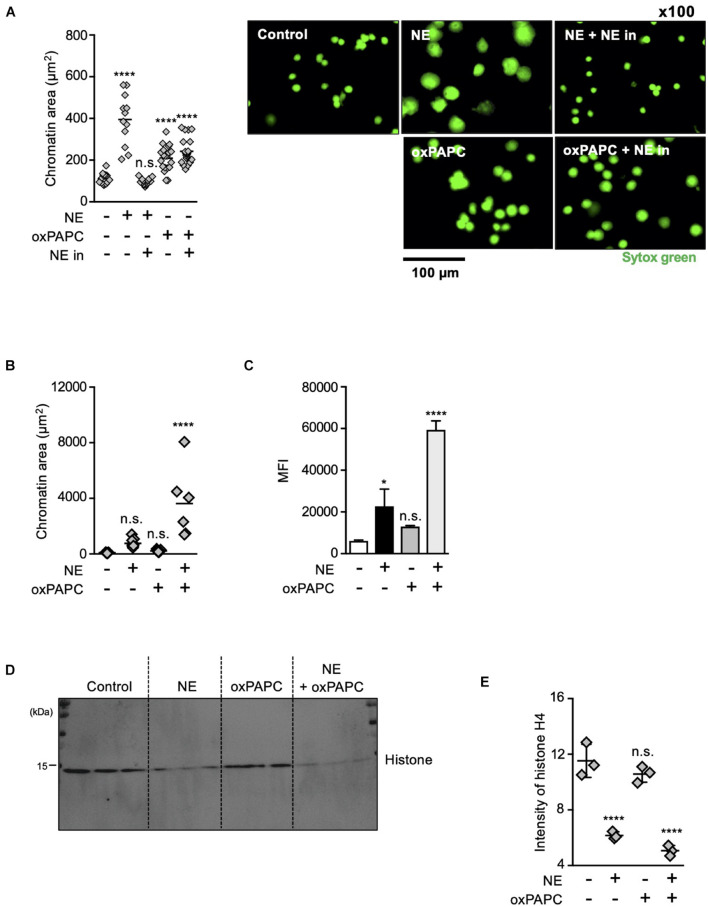
Oxidized phospholipids play a critical role in NET formation via NE- independent pathway. **(A)** Evaluation of chromatin decondensation ability of oxPAPC. Nuclei isolated from HL-60 cells were incubated in PBS or 5 μg/mL NE or 200 μg/mL oxPAPC for 60 min at 37°C and stained with SYTOX Green. Chromatin areas was quantified using Image-J software. Average values and s.d. of twelve nuclei in a single experiment are shown. ****P < 0.001, n.s., not significant, one-way ANOVA, compared with non-treated nuclei. Data are representative of two independent experiments (left). Representative images of fields from two experiments are shown (right). **(B,C)** Synergistic effects of NE and oxPAPC on chromatin decondensation. **(B)** Nuclei isolated from HL-60 cells were incubated in buffer, NE alone, oxPAPC alone, or NE + oxPAPC for 120 min at 37°C and stained with SYTOX Green. Chromatin areas was quantified using Image-J software. Average values and s.d. of 6–15 nuclei in a single experiment are shown. *P < 0.01, one-way ANOVA. Data are representative of two independent experiments. **(C)** Nuclei isolated from HL-60 cells were incubated in buffer, NE alone, oxPAPC alone, or NE + oxPAPC for 120 min at 37°C. The amount of DNA in supernatant was quantified as described in the Section “Materials and Methods.” Average values and s.d. of triplicate samples in a single experiment are shown. *P < 0.05, ****P < 0.001, n.s., not significant, one-way ANOVA, compared with non-treated nuclei. Data are representative of two independent experiments. **(D,E)** Evaluation of histone degradation by oxPAPC. Isolated nuclei were incubated with NE alone, oxPAPC alone, or NE + oxPAPC for 120 min at 37°C. The amount of histone H4 in each sample was analyzed by Western blotting. **(E)** Average values and s.d. of triplicate samples in a single experiment are shown. ****P < 0.001, n.s., not significant, one-way ANOVA, compared with non-treated nuclei. Data are representative of two independent experiments.

The results described above indicate that both oxidized phospholipids and NE are involved in NET formation. To explore how these two kinds of molecules contribute to NET formation, we analyzed the effects of these molecules on chromatin decondensation by using isolated nuclei. Incubation of isolated nuclei with recombinant NE resulted in significant nuclear swelling by measuring chromatin aria stained with SYTOX Green, and this morphological change was canceled by the NE inhibitor (Figure 6A). The addition of oxPAPC to the isolated nuclei also caused nuclear swelling, but the NE inhibitor had no effect on this oxPAPC-induced event (Figure 6A), indicating that oxPAPC induced nuclear swelling in a NE-independent manner. Furthermore, when NE and OxPAPC were combined, the decondensation of chromatin was promoted and the amount of DNA in the supernatant was increased (Figures 6B,C). NE is reported to contribute to the decondensation of chromatin by degrading histones (Papayannopoulos et al., 2010). Thus, we next examined whether OxPAPC had any effects on degradation of histones. As shown in Figures 6D,E, the addition of oxPAPC alone to the isolated nuclei did not have any effects on degradation of histone H4. However, further degradation of histone H4 was observed when the isolated nuclei were treated with NE and OxPAPC, suggesting oxPAPC has promoting effects on degradation of histone H4 by NE.

From these results, we conclude that oxidized phospholipids and NE coordinately play critical roles in chromatin decondensation.

## Discussion

In this study, we clearly demonstrated that lipid peroxidation is involved in NETosis and NET formation induced by PMA or ICs, which are the common inducers of these phenomena. Lipid peroxidation also plays a critical role in the execution of ferroptosis (Yang and Stockwell, 2016). However, the profiling of NETosis with ferroptosis inhibitors suggested that the mechanisms of lipid peroxidation in NETosis are distinct from those in ferroptosis. In particular, the minimal effects of an iron chelator on NETosis indicate that the oxidation of phospholipids takes place in an iron-independent manner in NETosis (Figure 1A). Here, we showed that MPO plays a crucial role in the lipid peroxidation during NETosis. Studies of MPO-deficient neutrophils in human or using MPO inhibitors clearly demonstrated that MPO is required for NETosis and NET formation (Papayannopoulos et al., 2010; Metzler et al., 2011, 2014). However, details of the roles of MPO remain an enigma. Metzler et al. (2014) reported that MPO plays a role in chromatin decondensation in cooperation with NE, and the enzymatic activity of MPO is not required for this effect. The same group also demonstrated that MPO plays a critical role in NE release from azurophilic granules during NETosis, and that MPO enzymatic activity may not be essential for this NE release. On the contrary, numerous studies of MPO inhibitors concluded that MPO enzymatic activity is indispensable for the execution of NETosis and NET formation (Papayannopoulos et al., 2010; Metzler et al., 2011, 2014; Palmer et al., 2012; Neubert et al., 2018). In this study, we clearly showed that MPO is involved in NETosis and NET formation by oxidizing phospholipids, indicating the dual roles (enzymatic activity-dependent and -independent) of MPO in NETosis and NET formation. As NE is recognized as a key molecule in chromatin decondensation, which is an essential step in NET formation (Papayannopoulos et al., 2010; Metzler et al., 2014), NE inhibitors, which are expected to be therapeutic medicines for various diseases in which NET formation, are considered an exacerbating factor. In fact, NE inhibitors have considerable therapeutic effects on such diseases as atherosclerosis or tumor growth and metastasis (Cools-Lartigue et al., 2013; Warnatsch et al., 2015; Albrengues et al., 2018). In contrast to these studies, it is also reported that NE inhibition is not sufficient to suppress NET-mediated thrombosis (Martinod et al., 2016). In this study, we clearly showed that NE and oxidized phospholipids synergistically promote chromatin decondensation, suggesting that a combination of inhibition of phospholipid peroxidation and NE may be another possible approach for the therapy of NET-related diseases. In this study, we demonstrated that oxidized phospholipids play a critical role in execution of NETosis and NET formation by using trolox, a lipid-specific antioxidant. To clarify the role of oxidized phospholipids in NETosis and NET formation more clearly, development of specific antagonists against phospholipid peroxidation should be required.

Our findings, together with recent studies of ferroptosis, suggest that NETosis and ferroptosis are executed by a common pathway called enzyme-mediated phospholipid peroxidation, although the mechanisms for the generation of oxidized phospholipids are different from each other. In these two cell-death pathways, massive lipid peroxidation presumably causes membrane damage that results in loss of membrane integrity. Localization of oxidized lipids supports this possibility. In fact, an analysis of liposomes used as a cell membrane model clearly showed that the oxidation of lipids alters the characteristics of lipid membranes in terms of fluidity and lateral diffusion (Wong-Ekkabut et al., 2007), and causes the permeability of the lipid membranes to release contents from liposomes (Zhang et al., 1993). In spite of this common mechanism in ferroptosis and NETosis, the morphological features of the nucleus are distinct between these two types of cell death. Neutrophils exhibit nuclear swelling and chromatin decondensation to release web-like structures during NETosis (Wang et al., 2009), whereas no obvious changes in nuclear morphology are observed during ferroptosis (Li et al., 2020). This obvious difference argues the presence of specific mechanisms for nuclear collapse in NETosis. In this study, we showed that oxidized phospholipids and NE synergistically promote chromatin release from isolated nuclei, suggesting the distinct roles of these two molecules. NE is reported to exist at high concentrations in azurophilic granules in neutrophils. With the activation of neutrophils, NE is released from the azurophilic granules to cytoplasm. The released NE degrades F-actin to arrest actin dynamics and cleaves histones to promote chromatin decondensation (Metzler et al., 2014). In addition to NE, PAD4, an enzyme abundant in neutrophils, also contributes to chromatin decondensation during NETosis (Li et al., 2010). These NE or PAD4-dependent nuclear changes likely cause entropic nuclear swelling to release nuclear contents easily by lipid peroxidation-dependent damage of the nuclear membrane (Neubert et al., 2018).

In addition to the synergistic effects of these molecules on nucleus, there may be another reason why the release of nuclear contents is likely to occur in the structure of neutrophil nuclei. The nucleus of mature neutrophils exhibits a unique nuclear envelope protein profile. During neutrophil differentiation, the amount of lamin A/C is reduced in the nuclear envelope, thereby resulting in an increase of nuclear flexibility (Manley et al., 2018). This change may contribute to the increased susceptibility to breakdown of the nuclear envelope during NETosis and NET formation. In any case, the mechanisms and dynamics of oxidized phospholipid-mediated breakdown of nucleus should be analyzed further to understand more precisely the process of NETosis and NET formation.

## Materials and Methods

### Animals and Human Donor-Derived Peripheral Blood

C57BL/6J mice were purchased from CLEA Japan. MPO KO mice (C57BL/6 background) were kindly provided by Professor Aratani (2018). GPx4^flox/flox^ mice (Yoo et al., 2012) were purchased from Jackson Laboratory (Stock Number #027964). LysM-Cre mice, obtained from RIKEN Bioresource Center (RBRC02302), were crossed with GPx4^flox/flox^ mice to obtain mice lacking GPx4 expression in macrophages and neutrophils (LysM-Cre-GPx4^flox/flox^ mice). All experiments using mice were approved by the Tokyo University of Pharmacy and Life Sciences Animal Care Committee and performed in accordance with applicable guidelines and regulations. The use of healthy human donor-derived peripheral blood was approved by the human ethics committee of Tokyo University of Pharmacy and Life Sciences. All methods were performed in accordance with the ethical guidelines for medical and health research involving human subjects in Japan. All blood donors gave informed consent.

### Reagents

Phorbol 12-myristate 13-acetate (PMA), anti-human albumin antibody, human serum albumin, dimethyl sulfoxide (DMSO), LPS (O111:B4), 4,4′-diaminodiphenyl sulfone (DDS), (±)-6-hydroxy-2,5,7,8-tetramethylchromane-2-carboxylic acid (trolox) 2-hydrazinobenzothiazole (SC002), 1-(6-methoxy- benzo[d] thiazol-2-yl)hydrazine (SC007), (5-chloro-2-methoxy- phenyl)hydrazine hydrochloride (SC008) and 1-[5-chloro-3-(trifluoromethyl)-2-pyridyl]hydrazine (SC009) were purchased from Sigma. Ionomycin and TBARS Assay Kit were purchased from Cayman. Anti-NE antibody and anti-histone H3 (citrulline R2 + R8 + R17) antibody were purchased from Abcam. Polyclonal rabbit anti-human MPO was purchased from DAKO. Anti-human/mouse MPO antibody was purchased from R&D Systems. Anti-GAPDH mAb HRP-Direct was purchased from MBL. Elastase from human neutrophils and MPO from human polymorphonuclear leukocytes were purchased from Merck. 1-Palmitoyl-2-arachidonoyl-sn-glycero-3-phosphocholine (PAPC) was purchased from Avanti Polar Lipids. Quant-iT^TM^ PicoGreen dsDNA Assay Kit, SYTOX^TM^ Green Nucleic Acid Stain, SYTOX^TM^ Orange Nucleic Acid Stain, and BODIPY 581/591 C11 were purchased from Thermo Fisher Scientific. 4′,6-Diamidino-2-phenylindole (DAPI) was purchased from DOJINDO. TMB Peroxidase Substrate was purchased from SeraCare Life Sciences. 2-Hydrazino-4-(trifluoromethyl)pyrimidine (SC001), 1-naphthalenyl hydrazine hydrochloride (SC004), 2-hydrazinoquinoline (SC005), phthalic hydrazide (SC014) and 4-phenylurazole (SC017) were purchased from Tokyo Chemical Industry. 2-Hydrazinobenzothiazole (SC002), 1-(6-methoxybenzo[d]thiazol-2-yl)hydrazine (SC007), (5-chloro-2-methoxyphenyl)hydrazine hydrochloride (SC008) and 1-[5-chloro-3-(trifluoromethyl)-2-pyridyl]hydrazine (SC009) were purchased from Merck. Fluoren-9-ylidene-hydrazine (SC003) and 2-hydrazinopyridine (SC006) were purchased from Wako. Ethyl 2-(phenylcarbamoyl)hydrazine-1-carboxylate (SC015), 1-methyl-4-phenyl-1,2,4-triazolidine-3,5-dione (SC 016) and 2-methyl-2,3-dihydrophthalazine-1,4-dione (SC018) were synthesized according to previously reported procedure (Sato et al., 2015, 2018). IBS013326 compounds were provided by the Chemical Biology Screening Center of Tokyo Medical and Dental University (Tokyo, Japan).

### Cell Preparation

To prepare mouse neutrophils, mouse bone marrow (BM) cells were isolated from femurs and tibias of WT mice, and peritoneal excluded cells were obtained from LysM-Cre-GPx4^flox/flox^ mice injected intraperitoneally with 3% thioglycolate. The cells were incubated with Fc blocker (2.4G2; Biolegend) and stained with biotinylated anti-Ly-6G (RB6-8C5; Biolegend) antibody. The cells were then incubated with anti-biotin-microbeads (Miltenyi Biotech). Ly-6G^high^ cells were enriched by magnetic sorting. The purity of the isolated mouse neutrophils was higher than 95% when assessed by flow cytometry (FACSVerse; BD Bioscience). To prepare human peripheral blood neutrophils, peripheral blood was collected from healthy adult volunteers using heparin. Red blood cells were removed using HetaSep^TM^ (STEMCELL Technologies) sedimentation according to the manufacturer’s protocol. Then, the cells were washed twice with RPMI1640 medium and further fractionated on a discontinuous Percoll PLUS (GE-Healthcare) gradient that consisted of layers with densities of 75%, 65%, and 55%. After the mixture was centrifuged for 30 min at 500 × *g*, the interface between the 65% and 75% layers was collected and washed twice with RPMI1640 medium. All procedures were conducted at room temperature. The preparations contained more than 95% of CD15^+^CD16^+^ neutrophils according to flow cytometric analysis. Cell viability was >98% according to trypan blue exclusion assays.

### Cell Culture and Differentiation of HL-60

HL-60 cells were purchased from RIKEN BioResource Center. The cells were maintained in RPMI-1640 medium supplemented with 10% FBS and 1% penicillin–streptomycin at 37°C in 5% CO_2_. To differentiate into neutrophil-like cells, HL-60 cells were incubated in RPMI-1640 medium supplemented with 1.25% DMSO, 10% FBS, and 1% penicillin–streptomycin at 37°C with 5% CO_2_ for 6–8 days.

### Generation of MPO- and NE-Deficient HL60 Cells Using CRISPR/Cas9 System

Human *MPO* and *ELANE* were targeted for CRISPR/Cas9-mediated disruption using sgRNA sequences generated by CRISPR direct^1^. Sequences were designed for use in the pLentiCRISPRv2GFP plasmid (Addgene #82416) and the oligo DNA was purchased from Thermo Fisher Scientific. The sequences are: human *MPO* (forward: 5′-CACCGTTGTTGCACATCCCGGTGA-3′; reverse: 5′-AAACTC ACCGGGATGTGCAACAAC-3′) and human *ELANE* (forward: 5′-CACCGGAAAAGACACGCGAGTCGG-3′; reverse: 5′-AA ACCCGACTCGCGTGTCTTTTCC-3′). These sequences were each cloned into pLentiCRISPRv2GFP following the depositor’s protocol. To produce lentiviral particles, HEK293T cells (2.2 × 10^6^ cells) were seeded in a 60 mm dish and incubated in D-MEM medium supplemented with 10% FBS and 1% penicillin–streptomycin at 37°C in 5% CO_2_. The cells were transfected with pLentiCRISPRv2GFP with human *MPO* or *ELANE* gRNA inserted, psPAX2, and pMD2.G (Addgene) using Lipofectamine 3000 (Thermo Fisher Scientific). The cells were incubated overnight. After changing to D-MEM medium supplemented with 10% FBS and 1% BSA, the cells were cultured for 60 h. The culture supernatant was centrifuged at 3,000 × *g* for 10 min at 4°C, filtered on a Millex-HV filter 0.45 μm (Merck), and used for transduction. Lentiviral particles were stored at −80°C until use. Transduction of HL-60 cells with the lentiviral particles was performed in 6-well plates. The lentivirus particles (2 mL) were added to 1 × 10^6^ cells in 2 mL of RPMI1640 medium supplemented with 10% FBS and 1% penicillin–streptomycin, followed by centrifugation at 1,200 × *g* at room temperature for 2 h. The cells were incubated overnight and GFP^+^ cells were sorted using an SH-800 cell sorter (SONY). The sorted GFP^+^ HL-60 cells were cloned by limiting dilution. Western blot analysis confirmed the absence of expression of the MPO or NE protein in all clones.

### Western Blot Analysis

To detect protein levels of MPO and NE, the cloned HL-60 cells (1 × 10^6^ cells) were washed with PBS and lysed with RIPA buffer (1 mM PMSF/1 μM pepstatin/1 μM leupeptin/50 mM Tris-HCl pH 8.0/150 mM NaCl/0.5% sodium deoxycholate/1% NP-40). The cell lysate was centrifuged at 4°C for 20 min at 20,000 × *g*. Proteins in SDS sample buffer were loaded on SDS-polyacrylamide gel electrophoresed, separated, and transferred onto PVDF membranes. The membrane was blocked with 5% skim milk/PBST (PBS/0.2% Tween 20) for 1 h. Rabbit polyclonal anti-MPO antibody (Dako) or rabbit monoclonal anti-NE antibody (Abcam) were reacted as the primary antibody. After washing with PBST, goat HRP-anti-rabbit IgG antibody (Dako) was reacted as the secondary antibody. After washing with PBST, SuperSignal West Pico PLUS Chemiluminescent Substrate (Thermo Fisher Scientific) was reacted, and the signal was detected with LAS4000mini (GE Healthcare).

To detect protein levels of citH3 in lungs, lungs (100 mg) in 1 mL RIPA buffer with protease inhibitors were homogenized using a homogenizer (Bioprep-6, Allsheng, Hangzhou, China) at 3800 rpm, 2 cycles, and 30 s. The samples were centrifuged at 20,000 × *g* for 10 min at 4°C, and protein concentration of the supernatants was determined using a Bicinchoninic Acid (BCA) Protein Assay Kit (#23225, Thermo Fisher Scientific). Proteins (30 μg) in SDS sample buffer were loaded on SDS-polyacrylamide gel electrophoresed, separated, and transferred onto PVDF membranes. The immunoblots were incubated in blocking buffer [5% skim milk in phosphate-buffered saline (PBS) with 0.1% Tween 20 (PBST)] for 60 min at room temperature and probed with anti-citH3 (#ab5103, Abcam) or anti-GAPDH mAb-HRP-DirecT (#M171-7, Medical & Biological Laboratories) overnight at 4°C. Then, for detection of citH3, the immunoblots were washed three times for 5 min with PBST, incubated with polyclonal goat anti-rabbit IgG-HRP (#P0448, Dako) for 30 min at room temperature in blocking buffer, and washed three times with PBST again. Immunodetection was performed using a SuperSignal^TM^ West Pico PLUS Chemiluminescent Substrate (#34580, Thermo Fisher Scientific).

### MPO and NE Enzymatic Activity Assay

HL-60 cells (0.4 or 1 × 10^6^ cells) were washed with PBS and lysed with 100 μL lysis buffer (PBS/0.2% Triron X-100 for MPO enzymatic activity assay or PBA/1% Triron X-100 for NE enzymatic activity assay). The cell lysate was centrifuged at 4°C for 20 min at 20,000 × *g*. the protein concentration of the supernatant was measured by the Pierce BCA Protein Assay Kit (Thermo Fisher Scientific). To evaluate MPO enzymatic activity, TMB Microwell Peroxidase Substrate System was added to the cell lysate (25 μg/mL or 125 μg/mL) and the reaction was allowed to proceed for 10 min at room temperature in the dark. Absorbance at 650 nm was measured by SH-9000 Lab (CORONA). To evaluate NE enzymatic activity, 1 mg/mL cell lysate was reacted with an Elastase Substrate, Fluorogenic (MeOSuc-AAPV-AFC) (ab142178) (Abcam), and the fluorescence intensity at 380/500 nm was measured every 2 min for 20 min at 37°C with SH-9000Lab. ΔRFU was calculated using the following formula [ΔRFU 380/500 nm = (RFU2-RFU2BG) – (RFU1-RFU1BG)]. The fluorescence intensity after 0 min was used for RFU1 and the fluorescence intensity after 20 min was used for RFU2. RFUBG is the background value. IC_50_ value was evaluated by GraphPad Prism software.

### Construction of MPO Overexpressing HL-60

To obtain human *MPO* cDNA, total RNA was extracted from HL-60 cells using an RNeasy Mini Kit (QIAGEN). Reverse transcription was performed by ReverTra Ace^®^ qPCR RT Master Mix (TOYOBO). PCR was performed using reverse transcription products as a template to amplify MPO cDNA. The primer sequences are (forward, 5′-CGGGATCC GATTGAGCAGCCCAGGAGAA-3′, reverse, 5′-CCGCTCGA GCTAGGAGGCTTCCCTCCAGGAAGCCAGGTTCAA-3′).

To avoid genome editing against exogenous *MPO* cDNA by the gRNA described above, the mutant *MPO* cDNA, in which mutations were introduced into sequences corresponding to gRNA without any changes in amino acid sequences was generated by site-directed mutagenesis by PCR (Ho et al., 1989) with human *MPO* cDNA as a template. The primer sequences are (Mutation points are underlined: forward, 5′-CGGGATCC GATTGAGCAGCCCAGGAGAA-3′, reverse, 5′-CCGCTCGAG CTAGGAGGCTTCCCTCCAGGAAGCCAGGTTCAA-3′, for- ward, 5′-TTACTGGAATGTGTAATAATAGACGCAGCCCCAC GCTGGG-3′, reverse, 5′-ATTATTACACATTCCAGTAATGGT GCGGTATTTGTCCTGC-3′). The cDNA was cloned into the *Bam*HI and *Xho*I site of pMXs-puro (pMXs-puro-hMPO1). pMXs-puro-hMPO1 was used as the expression plasmid for WT (exzyme-active) MPO.

To express enzymatic-inactive MPO, a point mutation which causes the mutation of aspartic acid to alanine mutation at amino acid position 96 (D96A) in the light chain (Shin et al., 2001) was inserted by site-directed mutagenesis by PCR using pMXs-puro-hMPO1 as a template. The primer sequences are (mutation points are underlined: forward, 5′-CGGGATCCGATTGA GCAGCCCAGGAGAA-3′, reverse, 5′-CCGCTCGAGCTAGGA GGCTTCCCTCCAGGAAGCCAGGTTCAA-3′ forward, 5′- AA TGGGGCCAGCTGTTGGACCACGCCCTCGACTTCACCCCT GAG-3′, reverse, 5′-CTCAGGGGTGAAGTCGAGGGCGTGGT CCAACAGCTGGCCCCATT-3′). The cDNA was cloned into the *Bam*HI and *Xho*I site of pMXs-puro (pMXs-puro mutant hMPO2).

To generate retroviral particles, PLAT-GP cells (2.2 × 10^6^, Cell Biolabs) suspended in D-MEM medium (10% FBS/1% penicillin–streptomycin) were seeded in a 60 mm dish and then incubated overnight. The cells were transfected with envelope vector pVSV-G, and pMXs-puro-hMPO1-or pMXs-puro mutant hMPO2 expression vector using FuGENE^®^ 6 Transfection Reagent (Promega) and then cultured for 48 h. The supernatant was collected, centrifuged at 3,000 rpm for 10 min at 4°C, and filtered using a Millex-HV filter 0.45 μm (Merck) to obtain the retrovirus solution. To concentrate retroviral vector, 32% PEG buffer [32% polyethylene glycol (Wako)/400 mM NaCl/40 mM HEPES (DOJINDO)] and the retrovirus solution were mixed at the ratio of 1:3. The mixture was centrifuged at 1,500 × *g* for 45 min at 4°C and the supernatant was removed. The pellet was diluted by PBS and used as the retrovirus solution. MPO-deficient HL-60 (5 × 10^4^ cells) suspended in RPMI-1640 medium (10% FBS/1% penicillin–streptomycin) was seeded on a 24-well plate. The retrovirus solution was added at a volume of 1:1 and centrifuged at room temperature at 2,000 × *g* for 2 h. The cells were cloned by limiting dilution. Western blot analysis confirmed the expression of the MPO protein in all clones.

### Preparation of Reagents

To prepare oxPAPC, PAPC in chloroform was evaporated to dryness in a glass tube and desiccated for at least 1 h *in vacuo*. PAPC was oxidized in an air incubator at 37°C for 48 h. OxPAPC was hydrated by adding serum-free medium (Advanced RPMI-1640; Thermo Fisher Scientific), vortexed at room temperature for 5 min, and then sonicated for 2 min. To prepare ICs, anti-human albumin antibody (Sigma) was diluted to 1 mg/mL by PBS and equal molecular of human serum albumin (Sigma) was added. The mixture was incubated at 37°C for 30 min. The experiment was carried out by diluting ICs to the concentration required in the experiment.

### NETosis Analysis

To analyze NETosis, human neutrophils, mouse neutrophils, or dHL60 cells (1.4 × 10^4^ cells) were seeded in a 96-well plate and stimulated with PMA, ionomycin, or ICs. Then, SYTOX Green (0.5 μM, Thermo Fisher Scientific) and Hoechst 33342 (1 μg/ml, Thermo Fisher Scientific) were added to the cells. After 30 min, the frequency of NETosis was measured by counting the number of SYTOX Green^+^ cells using an Operetta CLS (PerkinElmer).

### NETs Analysis

Isolated human neutrophils (1.4 × 10^4^ cells) were seeded in a 96 well plate and stimulated with PMA or ICs for 3.5 h. Then, DAPI was added to the cells. After 5 min, morphological analysis was performed using a fluorescence microscope (BZ-X710, Keyence).

### Morphological Analysis

For the morphological analysis of HL-60- or dHL-60 cells, cytospin slides were prepared by centrifuging the cells (4 × 10^4^ cells) in cytospin funnels at 1,000 rpm for 2 min using a Cytospin 4 (Thermo Fischer Scientific). The cells were stained with Diff-Quick Stain (Sysmex).

### BODIPY 581/591 C11 Analysis

To detect lipid peroxidation, 1.4 × 10^4^ neutrophils were seeded in round-bottom 96-well plates and stimulated with PMA, ionomycin, or ICs. 2 or 20 μM BODIPY 581/591 C11 (Thermo Fisher Scientific) was added to the cells for 30 min. Flow cytometric analysis was performed using BD FACSVerse.

To detect localization of oxidized BODIPY 581/591 C11, 1 × 10^5^ neutrophils were seeded in 35 mm glass bottom dish (Greiner Bio-One, No. 627975) and stimulated with 1 μM PMA and 200 μM DDS for 1.5 h. BODIPY 581/591 C11 (2 μM) was added to the cells for 30 min. Images were acquired using a Keyence BZ-X710 fluorescent microscope and images were analyzed using the BZ-X analyzer.

### TBARS Assay

Thirty micrograms of Egg PC (Avanti Polar Lipids) adjusted with PBS was incubated with recombinant MPO (R&D System) in the presence of 5 mM hydrogen peroxide for 60 min at 37°C. Then, lipid peroxidation of the sample was quantified using a TBARS Assay Kit (Cayman).

### Lipid Extraction From Neutrophils

Mouse neutrophils (5 × 10^6^ cells) were harvested in ice-cold methanol, and lipids were extracted using solid-phase extraction in a monospin C18 column (GL Sciences). PC (17:0/14:1), PE (17:0/14:1), PI (17:0/14:1), PS (17:0/14:1), and PG (17:0/14:1) were added at the final concentration of 100 nM each and used as the internal standard. The extracted lipids were reconstituted in 40 μL of chloroform: methanol = 1:2 and stored at −80°C until used.

### Wide-Targeted Analysis

A wide-targeted analysis was performed using an ACQUITY UPLC system (Waters) coupled with a triple quadrupole MS (QTRAP 6500, Sciex). The LC separation was per- formed using a reverse-phase column [ACQUITY UPLC HSS T3 (50 mm × 2.1 mm inner diameter, 1.8 μm particle size; Waters)] with a gradient elution consisting of mobile phase A (methanol/acetonitrile/water = 1:1:3 v/v/v containing 50 mM ammonium acetate and 10 nM EDTA) and mobile phase B (100% isopropanol containing 50 mM ammonium acetate and 10 nM EDTA). The LC gradient consisted of holding solvent (A/B:100/0) for 1 min, which was linearly converted into solvent A/B:50/50 for 4 min, linearly converted to solvent A/B:36/64 for 7 min, linearly converted to solvent A/B:5/95 for 1 min, and then held for 1 min. It was then returned to solvent A/B:100/0 and held for 5 min for re-equilibration. The injection volume was 3.5 μl, the flow rate was 0.350 mL/min, and the column temperature was 50°C. The multiple reaction monitoring (MRM) mode was used to quantify oxidized phospholipids in biological samples.

### Immunofluorescence Analysis

To detect the citrullination of histone H3 in mouse neutrophils or dHL60, 2.5 × 10^5^ cells were seeded in a 35-mm poly-_L_-lysine-coated glass bottom dish (MATSUNAMI) and stimulated with PMA, PMA + DDS, or ICs. The cells were then fixed with 4% paraformaldehyde for 10 min at room temperature and incubated in HBSS supplemented with 10% normal goat serum (Sigma), bovine serum albumin (Sigma) and 0.01% Tween 20 for 1 h for blocking. The cells were incubated first with hamster anti-histone H3 (citrulline R2 + R8 + R17) (citH3) antibody (11-11B-4F) (Yotsumoto et al., 2017) and then with anti-hamster IgG antibody coupled with Alexa Fluor 488 or 647 (Thermo Fisher Scientific). DNA was labeled using DAPI (DOJINDO).

To detect the citrullination of histone H3 in lungs, C57BL/6J mice were administrated PBS or LPS (50 μL at a concentration of 0.25 mg/ml) intranasally. After 24 h, the lungs were harvested and embedded in SCEM compound (SECTION-LAB). The cut surface was covered with adhesive film (Cryofilm type IIC9, SECTION-LAB, Japan) and frozen sections (5 μm) were prepared with a microtome (CM3050S, Leica Microsystems). The resulting sections were post-fixed with 100% EtOH for 10 s and 4% PFA/PBS(-) for 10 s, rinsed with PBS(-) for 20 s, and incubated with TN Blocking Buffer [0.1 M Trizma Base, pH 7.5, 0.15 M NaCl, 0.5% (w/v) blocking reagent (PerkinElmer, FP1020)] for 1 h at room temperature. The sections were then incubated with anti-citH3 antibody (Abcam, #ab5103, 1:250) or Human/Mouse MPO Antibody (R&D Systems, AF3667, 1:100) in TN blocking buffer for 1 h at room temperature. After three washes with PBS(-), the sections were incubated with Cy3 donkey anti-rabbit IgG (1:1000, Biolegend) or AlexaFluor488 donkey anti-goat IgG (1:1000, Jackson ImmunoReserch) in TN blocking buffer for 1hr in the dark at room temperature. After three washes with PBS(-), the sections were counterstained with DAPI, and the slides were mounted onto cover slips using mounting media (FluorSave Reagent, 345789, Merck Millipore).

### Flow Cytometric Analysis

For analysis of cell surface marker expression, the following Abs were used: anti-CD11b-PE (clone ICRF44) was purchased from BioLegend. Anti-CD16-APC (clone 3G8) was purchased from BD Biosciences. Flow data were collected using BD FACSVerse and analyzed using FlowJo X.

### Quantitative RT-PCR (qRT-PCR)

For the analysis of mRNA levels in HL-60 or dHL-60, RNA was extracted with a FavorPrep Total RNA Extraction Column (Favorgen) according to the manufacturer’s protocol. For qRT-PCR, cDNAs were synthesized using ReverTra Ace (TOYOBO). qRT-PCR was performed on cDNA with a THUNDERBIRD SYBR qPCR Mix (TOYOBO). Expression levels were normalized to 18s ribosomal RNA (rRNA). The following primer sequences were used for each gene: *PAD4* forward 5′-ACCAGAGCTGTGAAAGATCAGA-3′, reverse 5′- TCACAGTTCACCAGCAGGAT-3′; *NCF1* forward 5′-GTCGTG GAGAAGAGCGAGAG-3′, reverse 5′-TTCCGTCTCGTCAGG ACTGT-3′; *18s rRNA* forward 5′-CGGACAGGATTGACAG ATTG-3′, reverse 5′-CAAATCGCTCCACCAACTAA-3′.

### Analysis of Extracellular ROS Production

HL-60 or dHL-60 cells (7 × 10^3^ cells) were stimulated with 100 nM PMA in the presence of 200 μM lucigenin (Tokyo Chemical Industry). ROS release was monitored for 60 min at 37°C in a Microplate Luminometer (Berthold Technologies, LB96V).

### Analysis of Chromatin Decondensation Using Isolated Nuclei

For preparation of nuclei from HL-60 cells, the cells were lysed with 0.05% NP-40/PBS, followed by centrifugation at 1,400 rpm for 4 min at room temperature. The pellets containing nuclei were suspended in PBS. To analyze chromatin decondensation, isolated nuclei were incubated with NE and/or oxPAPC for 120 min at 37°C and stained with SYTOX Green. Chromatin areas was quantified using Image-J software. To analyze DNA release from isolated nuclei, isolated nuclei were incubated as described above, followed by incubation with 100 μU/ml DNase I (Worthington Biochemical) for 10 min. Then, the amount of DNA in supernatant was quantified using Quant-iT^TM^ PicoGreen^TM^ dsDNA Assay Kit (Thermo Fisher Scientific). To analyze degradation of histone H4, isolated nuclei were incubated as described above. The whole samples were diluted with the same volume of 2x RIPA buffer. The samples in SDS-sample buffer were loaded on SDS-polyacrylamide gel electrophoresed, separated, and transferred onto PVDF membranes. The membrane was blocked with 5% BSA/PBST (PBS/0.2% Tween 20) for 1 h. Rabbit polyclonal anti-histone H4 (Millpore) were reacted as the primary antibody. After washing with PBST, goat HRP-anti-rabbit IgG antibody (Dako) was reacted as the secondary antibody. After washing with PBST, SuperSignal West Pico PLUS Chemiluminescent Substrate (Thermo Fisher Scientific) was reacted, and the signal was detected with LAS4000mini (GE Healthcare).

### Statistical Analysis

Unpaired two-tailed Student’s *t*-tests was used to compare two groups. One-way ANOVA was used to compare multiple groups. All statistical analyses were performed using GraphPad Prism.

## Data Availability Statement

The raw data supporting the conclusions of this article will be made available by the authors, without undue reservation.

## Ethics Statement

The studies involving human participants were reviewed and approved by Human ethics committee of Tokyo University of Pharmacy and Life Sciences. The patients/participants provided their written informed consent to participate in this study. The animal study was reviewed and approved by Tokyo University of Pharmacy and Life Sciences Animal Care Committee.

## Author Contributions

TT contributed to the conceptualization, data curation, formal analysis, validation, investigation, methodology, writing–original draft, and writing–review and editing. AI, HS, ST, AY, RKA, RHA, MA, and TS contributed to the validation and investigation. SSa, YA, and SSh contributed to the resources. MT contributed to the conceptualization, supervision, funding acquisition, methodology, writing–original draft, project administration, and writing–review and editing. SY contributed to the conceptualization, data curation, formal analysis, supervision, funding acquisition, validation, investigation, methodology, writing–original draft, and writing–review and editing. All authors contributed to the article and approved the submitted version.

## Conflict of Interest

The authors declare that the research was conducted in the absence of any commercial or financial relationships that could be construed as a potential conflict of interest.

## Publisher’s Note

All claims expressed in this article are solely those of the authors and do not necessarily represent those of their affiliated organizations, or those of the publisher, the editors and the reviewers. Any product that may be evaluated in this article, or claim that may be made by its manufacturer, is not guaranteed or endorsed by the publisher.

## Footnotes

1
https://crispr.dbcls.jp

## References

[B1] Albrengues J. Shields M. A. Ng D. Park C. G. Ambrico A. Poindexter M. E. (2018). Neutrophil extracellular traps produced during inflammation awaken dormant cancer cells in mice. *Science* 361:eaao4227. 10.1126/science.aao4227 30262472 PMC6777850

[B2] Alflen A. Aranda Lopez P. Hartmann A. K. Maxeiner J. Bosmann M. Sharma A. (2020). Neutrophil extracellular traps impair fungal clearance in a mouse model of invasive pulmonary aspergillosis. *Immunobiology* 225:151867. 10.1016/j.imbio.2019.11.002 31761474 PMC7227411

[B3] Aratani Y. (2018). Myeloperoxidase: its role for host defense, inflammation, and neutrophil function. *Arch. Biochem. Biophys.* 640 47–52. 10.1016/j.abb.2018.01.004 29336940

[B4] Bjornsdottir H. Welin A. Michaelsson E. Osla V. Berg S. Christenson K. (2015). Neutrophil NET formation is regulated from the inside by myeloperoxidase-processed reactive oxygen species. *Free Radic. Biol. Med.* 89 1024–1035. 10.1016/j.freeradbiomed.2015.10.398 26459032

[B5] Clark S. R. Ma A. C. Tavener S. A. McDonald B. Goodarzi Z. Kelly M. M. (2007). Platelet TLR4 activates neutrophil extracellular traps to ensnare bacteria in septic blood. *Nat. Med.* 13 463–469. 10.1038/nm1565 17384648

[B6] Cools-Lartigue J. Spicer J. McDonald B. Gowing S. Chow S. Giannias B. (2013). Neutrophil extracellular traps sequester circulating tumor cells and promote metastasis. *J. Clin. Invest.* 123 3446–3458. 10.1172/jci67484 23863628 PMC3726160

[B7] Dixon S. J. Lemberg K. M. Lamprecht M. R. Skouta R. Zaitsev E. M. Gleason C. E. (2012). Ferroptosis: an iron-dependent form of nonapoptotic cell death. *Cell* 149 1060–1072. 10.1016/j.cell.2012.03.042 22632970 PMC3367386

[B8] Fuchs T. A. Abed U. Goosmann C. Hurwitz R. Schulze I. Wahn V. (2007). Novel cell death program leads to neutrophil extracellular traps. *J. Cell Biol.* 176 231–241. 10.1083/jcb.200606027 17210947 PMC2063942

[B9] Ho S. N. Hunt H. D. Horton R. M. Pullen J. K. (1989). Pease LR. Site-directed mutagenesis by overlap extension using the polymerase chain reaction. *Gene* 77 51–59. 10.1016/0378-1119(89)90358-2 2744487

[B10] Jorch S. K. Kubes P. (2017). An emerging role for neutrophil extracellular traps in noninfectious disease. *Nat. Med.* 23 279–287. 10.1038/nm.4294 28267716

[B11] Li J. Cao F. Yin H. L. Huang Z. J. Lin Z. T. Mao N. (2020). Ferroptosis: past, present and future. *Cell Death Dis.* 11:88. 10.1038/s41419-020-2298-2 PMC6997353 32015325

[B12] Li P. Li M. Lindberg M. R. Kennett M. J. Xiong N. Wang Y. (2010). PAD4 is essential for antibacterial innate immunity mediated by neutrophil extracellular traps. *J. Exp. Med.* 207 1853–1862. 10.1084/jem.20100239 20733033 PMC2931169

[B13] Magtanong L. Ko P. J. To M. Cao J. Y. Forcina G. C. Tarangelo A. (2019). Exogenous monounsaturated fatty acids promote a ferroptosis-resistant cell state. *Cell Chem. Biol.* 26 420–432.e429. 30686757 10.1016/j.chembiol.2018.11.016 PMC6430697

[B14] Malle E. Furtmuller P. G. Sattler W. Obinger C. (2007). Myeloperoxidase: a target for new drug development? *Br. J. Pharmacol.* 152 838–854. 10.1038/sj.bjp.0707358 17592500 PMC2078229

[B15] Manley H. R. Keightley M. C. Lieschke G. J. (2018). The neutrophil nucleus: an important influence on neutrophil migration and function. *Front. Immunol.* 9:2867. 10.3389/fimmu.2018.02867 30564248 PMC6288403

[B16] Martinod K. Witsch T. Farley K. Gallant M. Remold-O’Donnell E. Wagner D. D. (2016). Neutrophil elastase-deficient mice form neutrophil extracellular traps in an experimental model of deep vein thrombosis. *J. Thromb. Haemost.* 14 551–558. 10.1111/jth.13239 26712312 PMC4785059

[B17] Metzler K. D. Fuchs T. A. Nauseef W. M. Reumaux D. Roesler J. Schulze I. (2011). Myeloperoxidase is required for neutrophil extracellular trap formation: implications for innate immunity. *Blood* 117 953–959. 10.1182/blood-2010-06-290171 20974672 PMC3035083

[B18] Metzler K. D. Goosmann C. Lubojemska A. Zychlinsky A. Papayannopoulos V. (2014). A myeloperoxidase-containing complex regulates neutrophil elastase release and actin dynamics during NETosis. *Cell Rep.* 8 883–896. 10.1016/j.celrep.2014.06.044 25066128 PMC4471680

[B19] Neeli I. Khan S. N. Radic M. (2008). Histone deimination as a response to inflammatory stimuli in neutrophils. *J. Immunol.* 180 1895–1902. 10.4049/jimmunol.180.3.1895 18209087

[B20] Neubert E. Meyer D. Rocca F. Gunay G. Kwaczala-Tessmann A. Grandke J. (2018). Chromatin swelling drives neutrophil extracellular trap release. *Nat. Commun.* 9:3767. 10.1038/s41467-018-06263-5 PMC6138659 30218080

[B21] Palmer L. J. Cooper P. R. Ling M. R. Wright H. J. Huissoon A. Chapple I. L. (2012). Hypochlorous acid regulates neutrophil extracellular trap release in humans. *Clin. Exp. Immunol.* 167 261–268. 10.1111/j.1365-2249.2011.04518.x 22236002 PMC3278692

[B22] Papayannopoulos V. (2018). Neutrophil extracellular traps in immunity and disease. *Nat. Rev. Immunol.* 18 134–147. 10.1038/nri.2017.105 28990587

[B23] Papayannopoulos V. Metzler K. D. Hakkim A. Zychlinsky A. (2010). Neutrophil elastase and myeloperoxidase regulate the formation of neutrophil extracellular traps. *J. Cell Biol.* 191 677–691. 10.1083/jcb.201006052 20974816 PMC3003309

[B24] Pilsczek F. H. Salina D. Poon K. K. Fahey C. Yipp B. G. Sibley C. D. (2010). A novel mechanism of rapid nuclear neutrophil extracellular trap formation in response to *Staphylococcus aureus*. *J. Immunol.* 185 7413–7425. 10.4049/jimmunol.1000675 21098229

[B25] Rohm M. Grimm M. J. D’Auria A. C. Almyroudis N. G. Segal B. H. Urban C. F. (2014). NADPH oxidase promotes neutrophil extracellular trap formation in pulmonary aspergillosis. *Infect. Immun.* 82 1766–1777. 10.1128/iai.00096-14 24549323 PMC3993456

[B26] Rohrbach A. S. Slade D. J. Thompson P. R. Mowen K. A. (2012). Activation of PAD4 in NET formation. *Front. Immunol.* 3:360. 10.3389/fimmu.2012.00360 23264775 PMC3525017

[B27] Sato S. Hatano K. Tsushima M. Nakamura H. (2018). 1-Methyl-4-aryl-urazole (MAUra) labels tyrosine in proximity to ruthenium photocatalysts. *Chem. Commun. (Camb)* 54 5871–5874. 10.1039/c8cc02891e 29785428

[B28] Sato S. Nakamura K. Nakamura H. (2015). Tyrosine-Specific chemical modification with in situ hemin-activated luminol derivatives. *ACS Chem. Biol.* 10 2633–2640. 10.1021/acschembio.5b00440 26356088

[B29] Shin K. Hayasawa H. Lonnerdal B. (2001). Mutations affecting the calcium-binding site of myeloperoxidase and lactoperoxidase. *Biochem. Biophys. Res. Commun.* 281 1024–1029. 10.1006/bbrc.2001.4448 11237766

[B30] Wang Y. Li M. Stadler S. Correll S. Li P. Wang D. (2009). Histone hypercitrullination mediates chromatin decondensation and neutrophil extracellular trap formation. *J. Cell Biol.* 184 205–213. 10.1083/jcb.200806072 19153223 PMC2654299

[B31] Warnatsch A. Ioannou M. Wang Q. Papayannopoulos V. (2015). Inflammation. Neutrophil extracellular traps license macrophages for cytokine production in atherosclerosis. *Science* 349 316–320. 10.1126/science.aaa8064 26185250 PMC4854322

[B32] Wong-Ekkabut J. Xu Z. Triampo W. Tang I. M. Tieleman D. P. Monticelli L. (2007). Effect of lipid peroxidation on the properties of lipid bilayers: a molecular dynamics study. *Biophys. J.* 93 4225–4236. 10.1529/biophysj.107.112565 17766354 PMC2098729

[B33] Yang W. S. Stockwell B. R. (2016). Ferroptosis: death by lipid peroxidation. *Trends Cell Biol.* 26 165–176. 10.1016/j.tcb.2015.10.014 26653790 PMC4764384

[B34] Yang W. S. SriRamaratnam R. Welsch M. E. Shimada K. Skouta R. Viswanathan V. S. (2014). Regulation of ferroptotic cancer cell death by GPX4. *Cell* 156 317–331. 10.1016/j.cell.2013.12.010 24439385 PMC4076414

[B35] Yipp B. G. Petri B. Salina D. Jenne C. N. Scott B. N. Zbytnuik L. D. (2012). Infection-induced NETosis is a dynamic process involving neutrophil multitasking in vivo. *Nat. Med.* 18 1386–1393. 10.1038/nm.2847 22922410 PMC4529131

[B36] Yoo S. E. Chen L. Na R. Liu Y. Rios C. Van Remmen H. (2012). Gpx4 ablation in adult mice results in a lethal phenotype accompanied by neuronal loss in brain. *Free Radic. Biol. Med.* 52 1820–1827. 10.1016/j.freeradbiomed.2012.02.043 22401858 PMC3341497

[B37] Yotsumoto S. Muroi Y. Chiba T. Ohmura R. Yoneyama M. Magarisawa M. (2017). Hyperoxidation of ether-linked phospholipids accelerates neutrophil extracellular trap formation. *Sci. Rep.* 7:16026. 10.1038/s41598-017-15668-z PMC5700140 29167447

[B38] Zhang D. Yasuda T. Okada S. (1993). A carboxyfluorescein-enveloping liposome as a physicochemical damage model of the biomembrane for the study of lipid peroxidation. *J. Clin. Biochem. Nutr.* 14 83–90. 10.3164/jcbn.14.83

