# Supplementary material for: Oxidized Phospholipids and Neutrophil Elastase Coordinately Play Critical Roles in NET Formation
Source: Front Cell Dev Biol. 2021 Sep 9;9:718586. doi: 10.3389/fcell.2021.718586 (PMC8458647; doi:10.3389/fcell.2021.718586)
Supplement: Supplementary file 1 [file Data_Sheet_1.PDF]

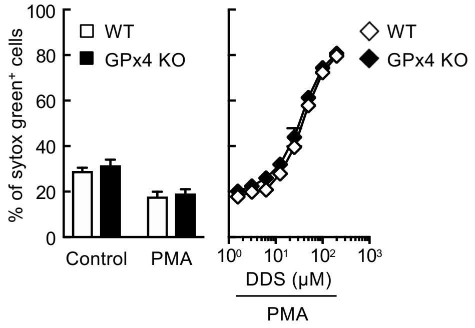

Figure S1. GPx4 is not involved in the regulation of NET induction. WT or LysM-Cre-GPx4$^{flox/flox}$ mice were injected peritoneally with 3% thioglycolate. Twenty-four hours later, neutrophils were purified from peritoneal excluded cell. Cells were stimulated with 1 μM PMA in the presence or absence of DDS (3-200 μM) for 2.5 hours. Cells were stained with SYTOX Green and Hoechst 33342. The proportion of NETosis was determined by counting the number of SYTOX Green^{+} cells using a high-content analysis system. Average values and s.d. of triplicate samples in a single experiment are shown. Representative data of two independent experiments are shown.

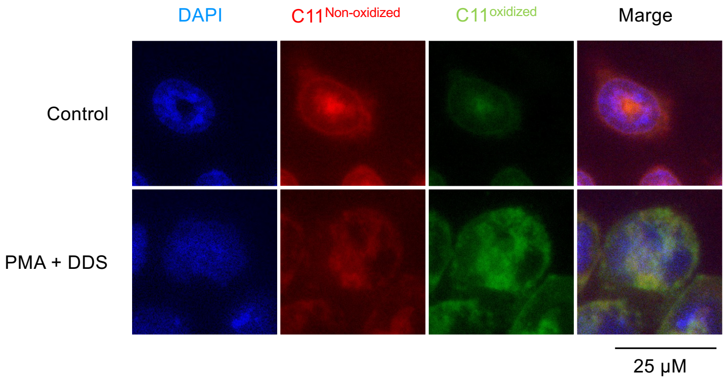

Figure S2. Oxidized lipids were accumulated in plasma membrane and around nuclear region of neutrophils undergoing NETosis. Mouse bone marrow neutrophils were stimulated with 1 μM PMA + 200 μM DDS for 1.5 hours. BODIPY 581/591 C11 (2μM) was added to the cells for 30 minutes. Images were acquired using a Keyence BZ-X710 fluorescent microscope and images were analyzed using the BZ-X analyzer. Representative images of two independent experiments are shown.

Figure. S1

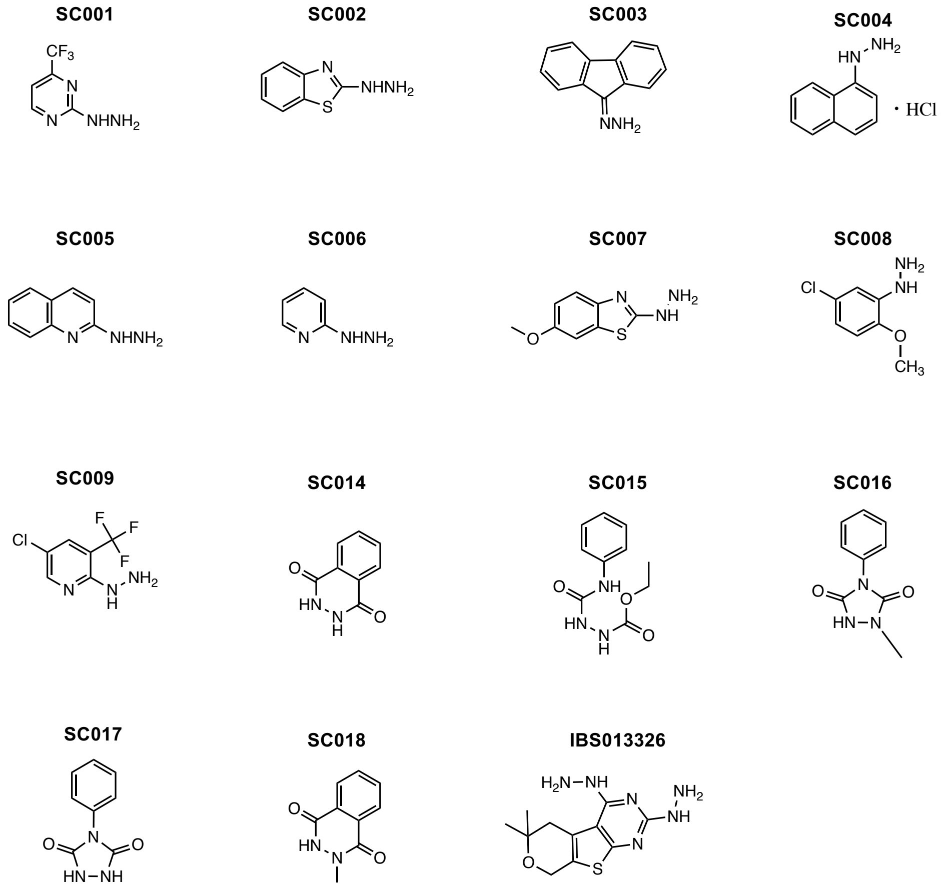

Figure S3. Structure of compounds containing hydrazine moiety.

T. Tokuhiro et al.

Figure. S2

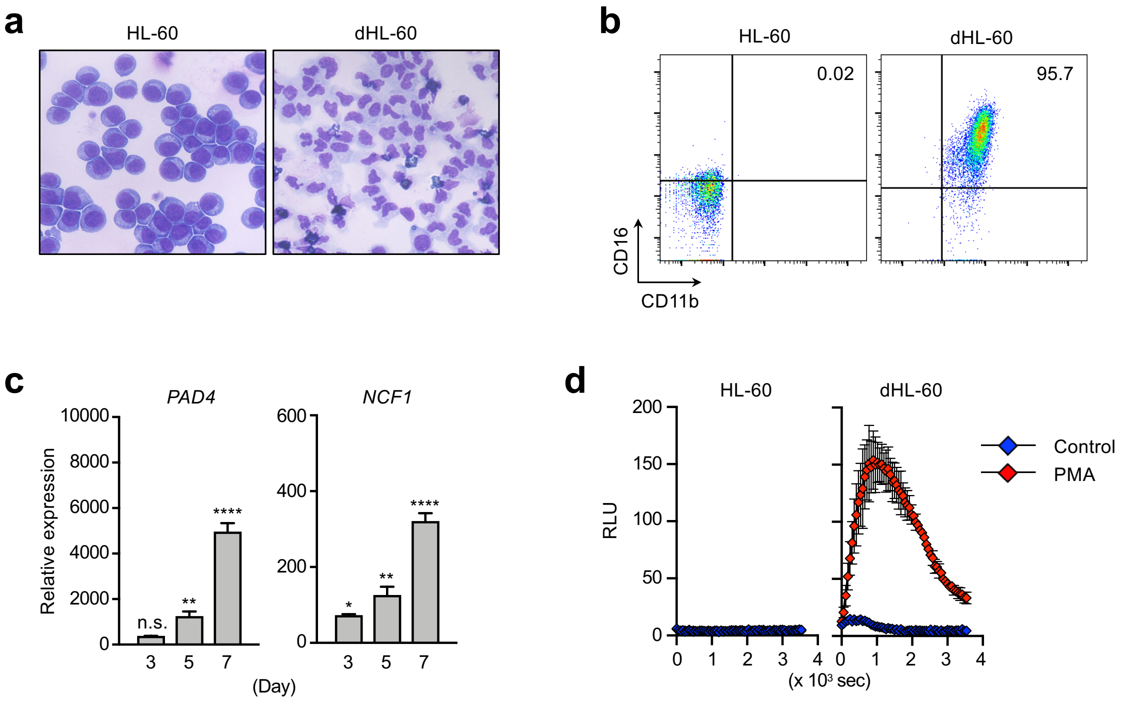

Figure S4. Characterization of DMSO-differentiated HL-60. (a–d) HL-60 cells were cultured for 7 days in the presence or absence of 1.25 % DMSO. The cells stained with Diff-Quick Stain Set. A representative images from two independent experiments are shown (a). Expression of surface CD11b and CD16 on HL-60 or DMSO-differentiated HL-60. Quadrants were established using appropriate isotype control antibodies. (B). mRNA expression levels of *PAD4* and *NCF1* were determined by qRT-PCR and are shown as fold change relative to that of DMSO-untreated HL-60. One-way ANOVA, *P < 0.05, **P < 0.01, ****P < 0.001, n.s., not significant (d). Extracellular ROS production of HL-60 or DMSO-differentiated HL-60 stimulated with 100 nM PMA as measured by the lucigenin-amplified chemiluminescence assay. Average values and s.d. of triplicate samples in a single experiment are shown. Data are representative of two independent experiments (d).

T. Tokuhiro et al.
